# Necrotizing Pancreatitis Following Acute Appendicitis: A Case Report of a Complex Clinical Course and Diagnostic Challenges

**DOI:** 10.7759/cureus.77556

**Published:** 2025-01-16

**Authors:** Bryan A Wallace, Maxime F Bariselle, Ellen A Wood, Mariam Ibrahim, Gabriela Marti, Yasmine Douidar, Feras Othman

**Affiliations:** 1 General Surgery, Delray Medical Center, Delray Beach, USA; 2 Medicine, St. George's University School of Medicine, True Blue, GRD; 3 General Surgery, University of Miami, Miami, USA

**Keywords:** acute appendicitis, complicated clinical course, diagnostic ct imaging, medical comorbidities, necrotizing pancreatitis

## Abstract

Necrotizing pancreatitis (NP) is a serious complication of acute pancreatitis. In this case report, we report a complex scenario in which a patient initially admitted for perforated appendicitis developed necrotizing pancreatitis during the hospital stay. Necrotizing pancreatitis is a rare clinical outcome, and by examining potential factors that contributed to this patient's condition, we offer insights into how to assess and potentially prevent future cases of necrotizing pancreatitis in similar patients.

An 83-year-old male with a complex past medical history, including coronary artery disease with 12 cardiac stents, obesity, hypertension, hyperlipidemia, and a prior pulmonary embolism, presented to the emergency department with acute-onset right lower quadrant abdominal pain, fever, and nausea. He was diagnosed with acute appendicitis with micro-perforations and underwent a laparoscopic appendectomy and lysis of adhesions. Postoperatively, the patient developed hypotension and tachycardia, requiring vasopressor support, as well as hematemesis and sepsis. A CT scan revealed a partially necrotic pancreas, prompting an exploratory laparotomy and pancreatic debridement. Blood cultures identified Candida glabrata, leading to the initiation of antifungal therapy. Despite aggressive critical care, including multiple abdominal surgeries, broad-spectrum antibiotics, and antifungal treatment, the patient developed multiorgan failure. On day 16, after going into cardiac arrest, the patient was pronounced deceased.

This case highlights the importance of early diagnostic strategies for high-risk patients with possible overlapping symptoms in the setting of acute abdominal pain. A broader scope of clinical assessments with timely intervention and aggressive management may be crucial in preventing severe outcomes, such as necrotizing pancreatitis.

## Introduction

Necrotizing pancreatitis (NP) is a progressive complication due to hypoperfusion of the pancreas during an episode of acute pancreatitis. NP can develop approximately 72 hours after the onset of acute pancreatitis symptoms, such as mild to severe epigastric pain, with possible radiation to the back [1,2,3]. A patient with acute pancreatitis should be considered for NP when computed tomography (CT) scans elicit fluid collections and non-enhancing regions of varying sizes within an enlarged pancreas [3]. NP can be classified as an acute necrotic collection or walled-off necrosis. Acute necrotic collections can typically form within 4 weeks of acute pancreatitis. Collections can be sterile or infected, with a CT-guided fine needle aspiration to distinguish the two. Walled-off necrosis of the pancreas typically forms after 8 weeks, in which the necrotic collection is contained within a well-defined wall [4,5,6]. The prognosis of NP without treatment is poor, as infection, hemorrhage, abdominal compartment syndrome, pancreatic duct disruption, and stricture formation can occur [5,7]. Resolution can be obtained with prompt recognition and treatment via the STEP-UP approach [5,6]. Undoubtedly, early recognition and treatment are vital for improved patient prognosis. Herein, we present a case of NP in a patient with a complicated clinical course. 

## Case presentation

On 7/31/2024, an 83-year-old Caucasian male, with a past medical history of obesity (BMI 33), coronary artery disease, hypertension, hyperlipidemia, restless leg syndrome, pulmonary embolism, and a past surgical history significant for 12 cardiac stents since 1998 was admitted for possible appendicitis. He presented to the emergency department (ED) for acute-onset abdominal pain localized to the right lower quadrant (RLQ) that started abruptly a few hours before his arrival to the ED. He described the pain as constant 7/10, exacerbated by consuming food, which prompted him to bring himself to the hospital. Associated symptoms included a low-grade fever and nausea, as well as slight cough, rhinorrhea, and shortness of breath, which were chronic as per family.

**Figure 1:**
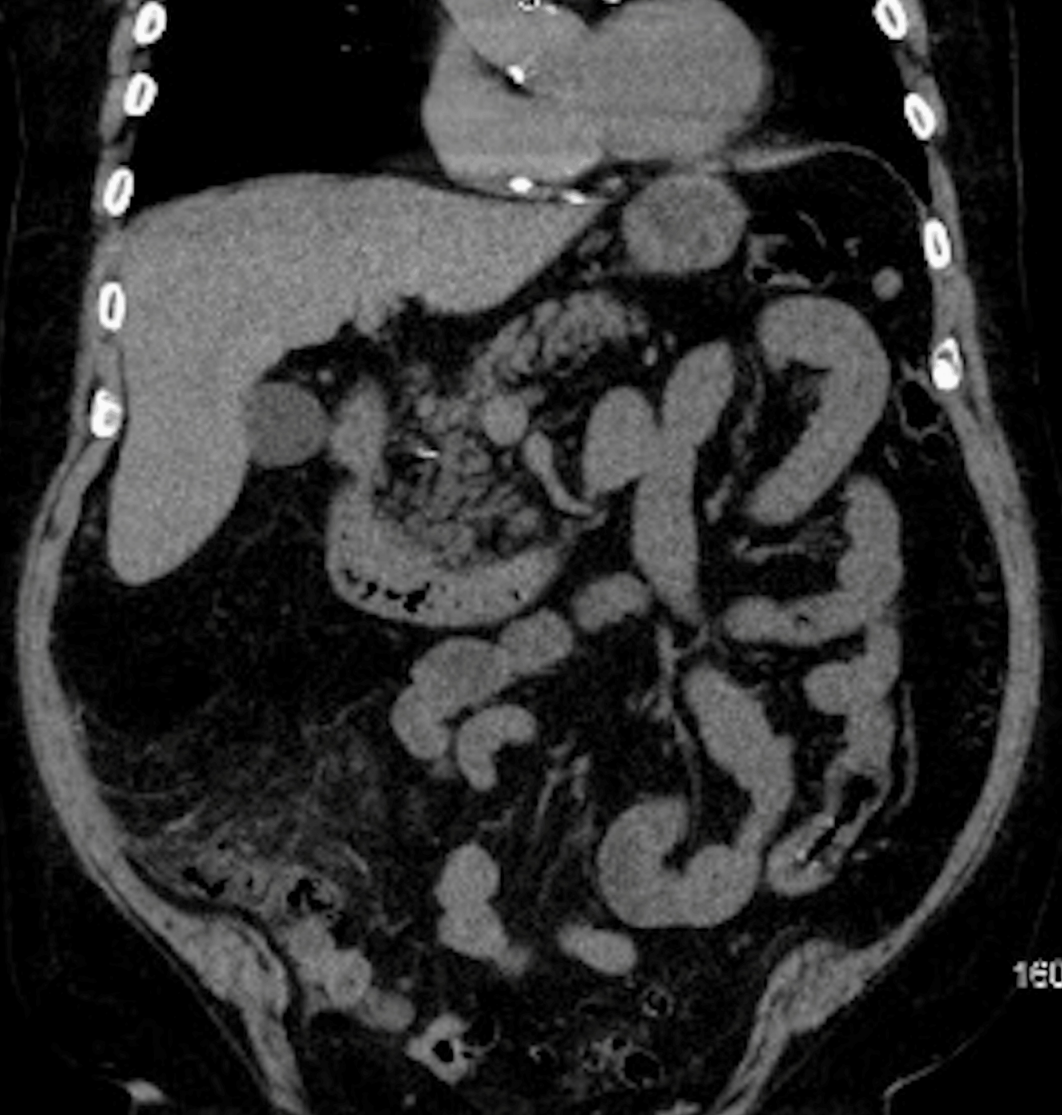
Abdominal CT-AP. CT-AP without contrast showing acute appendicitis with microperforations. AP: anteroposterior view, CT: computed tomography.

Upon a thorough physical exam, no erythema or cyanosis was seen; however, a sternal scar was noted. Bowel sounds were present upon auscultation. The abdomen was distended and diffusely tender on palpation, with a positive rebound tenderness in the RLQ. Due to the patient’s RLQ pain, fever, and leukocytosis, a CT scan was warranted, which confirmed acute appendicitis with micro-perforations (Figure 1). The patient was taken off his prescribed apixaban and was given IV piperacillin/tazobactam and IV normal saline. The patient was ultimately admitted for acute appendicitis and scheduled for operative management with a general surgery consultation. After a preoperative cardiac clearance, the patient was brought to the operating room (OR) for a successful laparoscopic appendectomy and lysis of adhesions around an umbilical hernia. While in the post-anesthesia care unit (PACU), the patient complained of persistent diffuse abdominal pain, localized primarily to the epigastric region. After stabilization of the patient in the PACU, he was brought to the cardiovascular intensive care unit (CVICU), where his vitals and abdominal pain were closely monitored.

**Figure 2:**
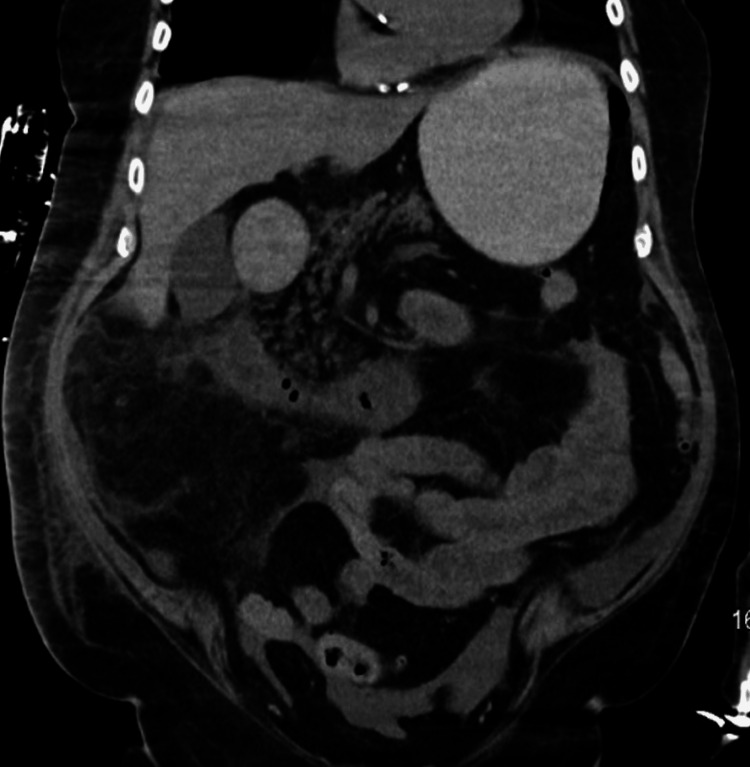
Abdominal CT-AP. CT-AP without contrast, showing free air and fluid surrounding the transverse colon, due to a possible perforated sigmoid diverticulitis vs post-surgical air and fluid. AP: anteroposterior view, CT: computed tomography.

**Figure 3:**
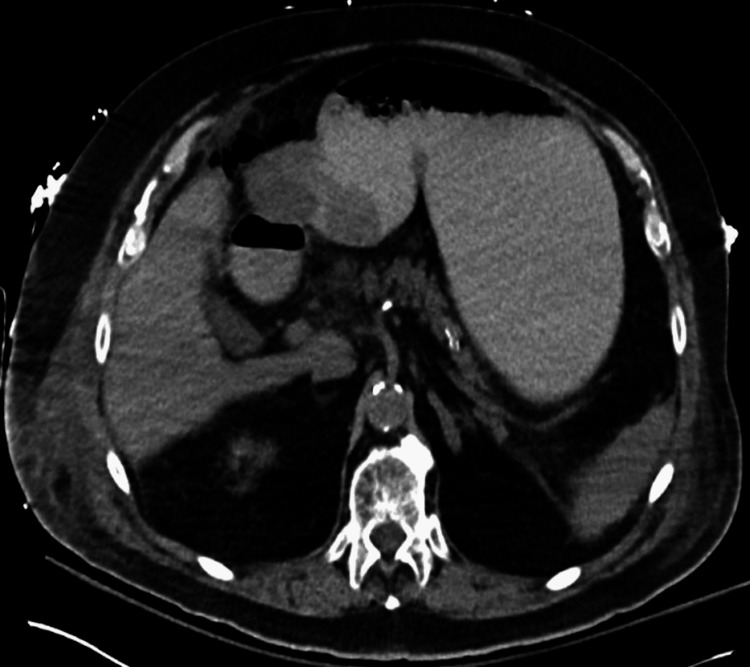
Abdominal CT-Axial. Axial without contrast, showing free air and fluid surrounding the transverse colon, due to a possible perforated sigmoid diverticulitis vs post-surgical air and fluid. CT: Computed tomography

Throughout the next 48 hours, while recovering in the CVICU, the patient had recurrent episodes of hypotension (systolic ranging from 64-97/diastolic 25-55 mmHg) and corresponding tachycardia, which required the use of vasopressors to maintain an optimal blood pressure. On day five of the patient’s hospitalization, the patient began developing hematemesis, out-putting two liters of bloody vomit. Within the same day, the patient’s condition deteriorated into cardiogenic shock, with his blood pressure at 77/55 mmHg, WBCs at 15.9x10^3^cells/mcL), and troponin I levels at 94.0 ng/mL. A rapid response code was activated, and the patient was placed on four vasopressors (epinephrine, norepinephrine, phenylephrine, and vasopressin) to stabilize the patient’s blood pressure. IV daptomycin was also initiated for broader antibiotic coverage. A CT without contrast was ordered for further evaluation of possible post-operative complications, which showed free air and fluid surrounding the transverse colon due to a possible perforated sigmoid diverticulitis vs post-surgical air and fluid (Figure 2, Figure 3). Due to the patient’s deteriorating condition along with free fluid around the transverse colon, the patient was promptly taken back to the OR for an exploratory laparotomy that revealed a partially necrotic pancreas, where the necrotized area was subsequently debrided. The abdomen was then washed out with 2L warm saline, an Abthera Wound Vac was placed, and the abdomen was left open. The patient was then returned to CVICU for critical care monitoring, where a nasogastric tube (NGT) and a Foley catheter were placed.

On day six, due to the patient’s unexplained hematemesis and persistently low hemoglobin (7.7 g/dL), a bedside esophagogastroduodenoscopy (EGD) was attempted to visualize the duodenum. During the procedure, the pylorus could not be visualized, and the duodenum could not be cannulated due to an accumulation of brown fluid in the stomach, which was presumed to be pancreatic in origin. Concurrently, the patient remained septic with a WBC reaching 20.2 x10^3^ cells/mcL. Blood cultures were then taken and were positive for *Candida glabrata*; additional IV Micafungin was added. The following day, the patient continued to remain in critical condition with no signs of improvement. Overnight, the patient’s vitals remained fluctuant, where labs were significant for upwards trending leukocytosis (peaking at 22.9x10^3^cells/mcL) and a downward trending hemoglobin (7.1 g/dL) (Table 1).

**Table 1:** Hemoglobin and WBC count trend throughout hospital stay. WBC: white blood cell, measured in 1x10^3^ cells per microliter (10^3^/mcL); normal WBC count: 4.0x10^3^ cells/mcL to 11.0x10^3^ cells/mcL; Hgb: hemoglobin, measured in grams per deciliter (g/dL); normal hemoglobin levels for an adult male: 13.5 g/dL to 17.5 g/dL Time: Military time.

Day	Time	WBC (x10^3^/mcL)	Hgb (g/dL)
July 31st	1:46	12.5	17.3
August 1st	6:37	13.6	15.8
August2nd	7:22	11.8	14.9
August 3rd	6:16	12.8	14.7
August 4th	0:30	9.4	16.5
August 5th	11:04	12.4	14.7
	19:27	15.7	14.2
	21:42	14	12.7
August 6th	0:06	16.3	10.9
	2:41	20.2	11
August 7th	2:48	21.4	9.6
	6:22	22.9	9.3
	9:28	22.9	9.3
	18:06	20.4	7.6
	23:33	-	8.5
August 8th	2:02	20	8.5
	10:34	-	8.7
	15:53	-	8.4
	23:03	-	8.2
August 9th	2:23	21.5	8.3
	10:35	-	7.7
	15:11	23.7	7.1
August 10th	0:12	-	8.6
	3:43	29.8	8.8
	17:10	-	7.4
	23:11	-	7.3
August 11th	6:10	28.3	9.2
	9:13	-	9.2
	14:45	29.3	8.5
August 12th	0:06	28.9	9.2
	3:07	27.7	8.6
	12:24	26.2	9.6
	16:06		8.9
	21:57	23.3	9.2
August 13th	0:17	-	8.8
	2:56	22.8	8.8
	6:16	-	9.1
	17:20	-	8.6
August 14th	0:15	20	8.5
	6:15	20.5	8.6
	8:15	-	8
	14:03	-	7.6
	19:58	-	7.2
August 15th	2:08	20.4	8.6
	8:23	-	7.4
	13:28	-	7.8
	15:48	18	7.8
	20:10	-	8.7
August 16th	2:27	21.2	9.1
	12:20	-	7.2

The following morning (day seven), the decision was then made to bring the patient back to the OR for a second pancreatic necrosectomy, abdominal washout, and intraoperative upper endoscopy. After partially debriding the necrotized pancreas, the pancreatic tissue was sent to pathology for a culture. During the same procedure, a leak test was also performed to visualize a possible perforation in the small bowel. Using saline while insufflating the stomach, an intraoperative endoscopy revealed air bubbles spotted within the duodenum, indicating a duodenal perforation. A Jackson-Pratt (JP) drain was then placed to drain the lesser sac and stomach. Within the next 24 hours that followed (day eight), the patient’s vitals were stable; however, the JP drain began draining serosanguineous fluid while the NGT suctioned frank blood.

As the patient remained comatose, going on nine days without eating, in addition to no significant improvement in his condition, the decision was made to return the patient to the OR for the fourth extensive surgical operation. On day nine, the patient underwent a third pancreatic necrosectomy, pyloric exclusion (where the pylorus of the stomach was stapled closed), as well as placements of a gastrostomy tube (for gastric drainage), jejunostomy tube (for feeding), and two peripancreatic drains. During the surgery, part of the omentum appeared necrotic, which was then debrided. Prior to the completion of the surgery, a myocutaneous advancement flap x2 was performed before placing another Abthera wound vac for delayed closure of the abdomen.

Throughout the following week, days 10-16 of hospitalization, the patient’s vitals remained stable with vasopressors. The peripancreatic drains continued to produce a dark brown fluid that developed into frank blood. At the same time, NGT and the G-tube continued to drain frank blood while the JP (from a lesser sac) drained serosanguineous fluid. On day 16, while still in the CVICU, the patient’s condition began to deteriorate once again and became hypotensive (88/38 mmHg). The patient went into multiorgan failure and eventually into cardiac arrest with no palpable pulses. Due to the patient having a DNR order in place, no resuscitation was attempted. The patient had succumbed to his ailments and was subsequently declared deceased.

## Discussion

We report a case of necrotizing pancreatitis (NP) where, on admission, the patient was diagnosed with acute appendicitis but developed severe complications and death due to advanced comorbidities and underlying inflammation of the pancreas. This patient presented with acute abdominal pain, fever, weakness, and shortness of breath. Although these symptoms are not specific, acute appendicitis was confirmed from an abdominal Computed tomography scan (CT scan), which revealed appendiceal inflammation with a micro-perforation (Figure 1). An urgent appendectomy was indicated and performed with no procedural complications. So, how did this case go from acute appendicitis to necrotizing pancreatitis, or was the diagnosis of acute pancreatitis overlooked on admission?

Acute pancreatitis is among the most common gastrointestinal-related conditions requiring hospital admissions in the United States [8-9]. Approximately 5-80 hospital admissions per 100,000 cases a year are due to acute pancreatitis. Of these acute pancreatitis cases, 20% lead to pancreatic necrosis, resulting in a mortality rate of 20-30% [8,10]. Necrotizing pancreatitis (NP) usually develops as a sequel to acute pancreatitis. Among other etiologies, acute pancreatitis is most commonly caused by gallstones and alcohol consumption [8-9]. One case report suggested that pancreatitis and appendicitis may be interconnected, as their patient presented with both diseases [6]. However, while acute appendicitis is widely known as the most prevalent abdominal surgery worldwide, there are no reports of acute appendicitis as an etiology of pancreatitis [11-12]. 

Acute abdominal pain often begins with nonspecific symptoms and gradually develops into more defined signs of a particular disease, making it challenging to accurately diagnose the underlying cause early on [13]. In particular, the early symptoms and presentation of acute pancreatitis and acute appendicitis can share clinical similarities, such as abdominal pain with nausea or vomiting [1,14]. Both pathologies can also be diagnosed via abdominal computed tomography scan (CT scan) [1,14]. However, as per the Atlanta Criteria, a diagnosis of acute pancreatitis requires two out of three of the following: abdominal pain consistent with pancreatitis; serum amylase or lipase three or more times the upper limit of normal; and findings consistent with pancreatitis on cross-sectional abdominal computed tomography (CT scan) [1,15]. In this case, a CT scan without contrast was used on admission, and the impression of the patient’s abdomen did not show any morphological abnormalities regarding the pancreas. This ruled out pancreatitis as the root cause of the abdominal pain, indicating no further workup was needed for amylase/lipase levels. 

Furthermore, studies have reported early unenhanced contrast CT scans of the abdomen are good prognostic indicators for acute pancreatitis and are highly effective in identifying pancreatic necrosis early on [16]. However, Kothari et al. conducted a study on the cost vs usefulness of CT scans when diagnosing Acute uncomplicated pancreatitis (AUP). Of the 405 patients who were previously clinically diagnosed with AUP, 210 patients underwent CT imaging with or without contrast, and 208 (99.05%) of the cases resulted in normal findings despite the use of contrast [17]. Although they are sensitive enough to predict the severity in a patient with classic pancreatitis symptoms, research suggests that patients who have clinical and biochemical evidence do not necessarily warrant a CT scan for the diagnosis [16-17]. As seen in this case, despite the use of an abdominal CT scan (Figures 1-3), acute pancreatitis was undetected, ultimately leading to complicated necrotizing pancreatitis. 

Necrotizing pancreatitis is primarily caused by acute pancreatitis and hypoperfusion, leading to severe inflammation that triggers autodigestion of pancreatic tissue [18]. As the damage progresses, pro-inflammatory mediators are released, potentially causing a systemic inflammatory response, hypotension, organ failure, and further pancreatic destruction [10,18]. Although acute pancreatitis is mostly a self-limiting disease, a minority of patients can progress to severe necrotizing pancreatitis. Parallel to the pathophysiology of NP, once necrosis sets in, local and systemic complications are common [19]. As noted by Ralapanawa et al. where a patient presented with an NSTEMI, which ultimately was due to an underlying necrotic pancreas [19]. Like so, postoperatively, on day five, our patient developed cardiogenic shock and an NSTEMI. The cause at the time was unknown, which prompted a stat CT scan without contrast, which showed pancreatic fluid within the stomach (Figures 2, 3). Due to the patient's hematemesis and hemodynamic instability, the patient was brought back to the OR for an exploratory laparotomy and abdominal washout, where the necrotic pancreas was identified and debrided. 

In cases like this one, where the patient is of advanced age, obese, and has an extensive cardiovascular history, the underlying cause of NP was determined to be from hypoperfusion of the pancreas. Further supporting this etiology is Yoshida et al., who reported a case of malignant hypertension complicated with necrotizing pancreatitis [2]. Despite being different pathologies, hypoperfusion was the exacerbating factor. 

This case highlights that given the similarities in presentation and the frequency of hospital diagnoses between pancreatitis and appendicitis, acute pancreatitis was not initially detected on clinical assessment. The possibility that early pancreatitis may not always be visible on unenhanced CT scans likely contributed to this oversight, which allowed the evolution of necrotizing pancreatitis. To prevent mortality, particularly in patients who are of high risk and advanced age like our patient, it may be recommended to broaden the clinical assessment on admission for patients presenting with abdominal pain. To ensure a timely diagnosis for any concurrent abdominal pathologies, incorporating a complete biochemical workup including amylase/lipase levels, routine inflammatory markers, and repeated imaging- may be essential. This approach may further reduce the risk of complications in such high-risk patients and allow the initiation of early aggressive management. Given the poor prognosis of cases like this one, additional retrospective studies and research is warranted to improve treatment strategies to optimize outcomes. 

## Conclusions

This case report emphasizes the challenges of diagnosing and managing necrotizing pancreatitis, particularly in patients with multiple comorbidities, such as cardiovascular disease and obesity. The progression from an initial diagnosis of acute appendicitis to the eventual development of NP underscores the need for heightened clinical suspicion and the use of advanced imaging in high-risk patients. Clinicians should be aware of the potential for concurrent abdominal pathologies, especially in complex cases, and consider more comprehensive diagnostic workups, including regular monitoring of biochemical markers and contrast-enhanced CT scans. This case highlights the need for a multidisciplinary approach to care, where timely interventions can significantly impact patient outcomes. Further research is warranted to refine diagnostic protocols and therapeutic strategies, ensuring that patients with necrotizing pancreatitis receive the most effective and timely care.
